# Early Clinical Outcomes of Definitive Volumetric Modulated Arc Therapy in Locally Advanced Oral Cavity Cancers: A Prospective, Single-Center Study

**DOI:** 10.7759/cureus.94432

**Published:** 2025-10-13

**Authors:** Sreenivasa Rao Bonala, Bala Venkat Subramanian, Mittimani Subhash, Selvamary Henita

**Affiliations:** 1 Radiation Oncology, Sri Venkateswara Institute of Medical Sciences, Tirupati, IND

**Keywords:** acute toxicity, cisplatin, definitive chemoradiotherapy, india, oral cavity squamous cell carcinoma, progression-free survival, tumor response, volumetric modulated arc therapy

## Abstract

Background

Evidence from prospective evaluations of outcomes in locally advanced, surgically unresectable oral cavity squamous cell carcinoma (OCSCC) treated with definitive chemoradiotherapy is limited.

Methodology

We conducted a single-center, prospective, observational study to evaluate early tumor response, acute toxicities, treatment compliance, and survival outcomes in patients with unresectable, locally advanced OCSCC treated with volumetric modulated arc therapy (VMAT) with concurrent three-weekly cisplatin in feasible patients.

Results

A total of 55 consecutive patients (median age = 56 years; Eastern Cooperative Oncology Group performance status = 0-2) were included. Overall, 40 (72.7%) patients received concurrent cisplatin-based chemoradiotherapy, and 15 (27.3%) underwent VMAT alone. At three months post-treatment, the objective response rate for the entire cohort was 78.2% (95% confidence interval (CI) = 65.0-88.2%), and the disease control rate was 90.9% (95% CI = 80.0-97.0%). Severe (grade ≥3) mucositis occurred in approximately half of the patients, whereas other acute and hematologic toxicities were generally ≤25%. The median overall treatment time was 43 days. With a median follow-up of 13.4 months, the median progression-free survival (PFS) and overall survival (OS) were 16.7 (95% CI = 14.9-18.5), and 18.6 months (95% CI = 16.0-21.2), respectively, with one-year PFS and OS rates of 78% (95% CI = 67-89) and 80% (95% CI = 69-91), respectively.

Conclusions

In patients with unresectable, locally advanced OCSCC with good performance status, definitive chemoradiotherapy delivered with VMAT is feasible and effective, achieving reasonable response rates and acceptable acute toxicities; however, longer follow-up is required to fully assess late toxicities.

## Introduction

Oral cancers represent a major public health burden worldwide, particularly in South and Southeast Asia, where the prevalence of tobacco and betel-quid use is high [1,2]. Despite advances in diagnosis and treatment, they are associated with substantial morbidity and mortality [1,2]. Oral cavity squamous cell carcinoma (OCSCC) constitutes the majority of these cancers. The standard of care for resectable OCSCC is surgical excision with appropriate reconstruction, followed by adjuvant radiotherapy with or without concurrent chemotherapy in the presence of high-risk pathological features [3,4]. Over the past two decades, the introduction of advanced radiotherapy techniques, such as intensity-modulated radiotherapy (IMRT) and volumetric modulated arc therapy (VMAT), has enabled more conformal dose delivery, improved normal tissue sparing, and potentially enhanced locoregional control. VMAT, in particular, combines dosimetric advantages with shorter beam-on time, which reduces overall treatment duration and minimizes intrafraction motion, thereby improving the therapeutic ratio in locally advanced head and neck cancers [5,6].

Nevertheless, a substantial proportion of patients present with disease that is technically unresectable, medically inoperable due to comorbidities, or decline surgery altogether [7]. Management of these patients remains challenging and varies according to tumor burden, performance status, and treatment intent, ranging from radical to palliative approaches [7-11]. Definitive chemoradiation is often employed for patients with a good performance status, drawing largely on evidence extrapolated from broader head and neck cancer trials [12,13].

However, prospective data specifically evaluating definitive radiotherapy for unresectable OCSCC are scarce. Most available evidence comes from retrospective, single-institution series [14-19], often using conventional techniques with heterogeneous patient populations and limited reporting of early clinical outcomes. To address this evidence gap, we conducted a prospective, single-center study evaluating definitive VMAT with or without concurrent cisplatin-based chemotherapy in a homogeneous cohort of patients with locally advanced, unresectable OCSCC. Our primary objective was to assess the early clinical outcomes, including response rates and survival. Our secondary objective was to evaluate the acute toxicity. By focusing on a standardized treatment approach, our study aims to provide contemporary data to inform best practices and strengthen the evidence base for the nonsurgical management of this challenging disease.

## Materials and methods

Study design and setting

This prospective, single-center, observational cohort study was conducted at the Department of Radiation Oncology, SVIMS Cancer Center, Sri Venkateswara Institute of Medical Sciences (SVIMS), Tirupati, India, between December 2023 and July 2025. The study complied with the Good Clinical Practice and the Declaration of Helsinki and received approval from the Institutional Ethics Board (Roc. No.: AS/11/IEC/SVIMS/2017; IEC No.: 1508, dated 02-11-2023) and was prospectively registered with the Clinical Trials Registry of India (CTRI/2024/02/062620).

Eligibility criteria

Consecutive patients were enrolled if they had newly diagnosed, histologically confirmed squamous cell carcinoma (SCC) of the oral cavity, were aged 18-75 years, had an Eastern Cooperative Oncology Group (ECOG) performance status of 0-2, and had stage III-IVB disease based on the eighth edition of the American Joint Committee on Cancer. All patients were deemed unsuitable for primary surgery (by surgical assessment or patient choice) but were fit for radical radiotherapy. The exclusion criteria were distant metastases and any prior radiotherapy or major surgery in the head and neck region.

Pretreatment workup

All patients underwent a standardized pre-treatment evaluation before starting therapy. This included a detailed history, physical examination of the oral cavity and neck, and assessment of the performance status. Baseline hematological and biochemical investigations (complete blood counts, renal and liver function tests, and electrolytes) were performed. Dental evaluation was performed, including necessary prophylactic extractions and oral hygiene counseling. Imaging included contrast-enhanced CT (CECT) of the head and neck and, where indicated, MRI for better delineation of soft tissue or perineural involvement. Chest imaging (X-ray or CT) and ultrasonography or CT of the abdomen were performed to exclude distant metastases. Nutritional assessment, speech and swallowing evaluation, and counseling on smoking and tobacco cessation were routinely provided. All patients received individualized supportive care plans and were medically optimized before initiating radiotherapy or chemotherapy.

Radiotherapy protocol

Patients were immobilized in the supine position using a five-point thermoplastic mask. CT simulation was performed on a dedicated simulator (Siemens Go.Sim, Germany) with intravenous contrast, and 3-mm slices were acquired from the vertex to the carina. The images were imported into the Monaco treatment planning system (TPS; Elekta AB, Stockholm, Sweden) for target delineation and treatment planning.

Gross tumor volume (GTV) was defined as visible disease on clinical examination and imaging studies. Primary clinical target volumes (CTVs) were delineated according to consensus guidelines [20], using a 5-mm expansion around the GTV for CTV1 and an additional 5-mm expansion for CTV2 to encompass areas at a high risk of microscopic spread. Grossly involved lymph nodes received a 5-mm expansion for nodal CTV1, extended to 8 mm in cases with extranodal extension. The selection and delineation of elective nodal volumes followed published guidelines [21,22]. All CTVs were edited to exclude air cavities and natural anatomic barriers, such as uninvolved bone or muscle.

CTV-high risk (HR) was derived by combining the primary CTV1 and nodal CTV1, whereas CTV-low risk (LR) was derived by combining the primary CTV2 and nodal CTV2. A uniform 5-mm margin was added to each CTV-HR and CTV-LR to generate the respective high-risk (PTV-HR) and low-risk (PTV-LR) volumes. Normal structures, including the parotid and submandibular glands, pharyngeal constrictors, mandible, spinal cord, brainstem, optic nerves, optic chiasm, and cochleae, were contoured according to international guidelines [23]. All target volume delineations, including GTV and CTV, were peer-reviewed by the senior radiation oncologist and unit head before treatment planning to ensure accuracy and consistency.

All patients received external beam radiotherapy using VMAT, with prescribed doses of 66 Gy (2.2 Gy per fraction) and 54 Gy (1.8 Gy per fraction) to the PTV-HR and PTV-LR, respectively, delivered over 30 fractions (five fractions per week) using a simultaneous integrated boost technique. The planning objectives included ≥95% coverage of the PTV by the prescribed dose with acceptable dose conformity and homogeneity, limiting the maximum dose to ≤107%, and avoiding cold spots while respecting established dose constraints for organs at risk [24]. Treatment was delivered on an Elekta Synergy linear accelerator (Elekta AB, Stockholm, Sweden), with daily cone-beam CT during the first three fractions and weekly thereafter to ensure image guidance and accurate position verification.

Concurrent chemotherapy

Concurrent cisplatin was planned for eligible patients who received definitive VMAT. Cisplatin was administered at 100 mg/m² every three weeks to patients aged ≤70 years with an estimated glomerular filtration rate (GFR) ≥60 mL/minute (Cockcroft-Gault formula) and ECOG performance status ≤1. For patients with GFR between 45 and 59 mL/minute, 75% of the calculated dose was administered. Cisplatin was omitted in patients aged >70 years, GFR <45 mL/minute, ECOG performance status 2, or in those with significant comorbidities or intolerance after the first cycle.

All patients receiving chemotherapy were premedicated with aprepitant, ondansetron, and dexamethasone. Potassium and sodium replacement were administered as required, along with intensive pre- and post-hydration using normal saline. Weekly complete blood count and renal function tests were performed during treatment. Chemotherapy was withheld in the event of grade ≥3 hematologic toxicity, renal impairment, or patient refusal.

Follow-up and outcome assessment

Patients were reviewed weekly during radiotherapy for acute toxicities, treatment compliance, and supportive care. After the completion of radiotherapy, follow-up was scheduled at six weeks, followed by every three months for the first year, and every six months thereafter. At each visit, a comprehensive clinical examination of the primary site and neck nodes was performed. Imaging with CECT or MRI of the head and neck was performed at three months post-treatment and thereafter, as clinically indicated.

Tumor response was evaluated using the Response Evaluation Criteria in Solid Tumors (RECIST) version 1.1 [25] based on clinical and radiological findings, and responses were categorized as complete response (CR), partial response (PR), stable disease (SD), or progressive disease (PD). The objective response rate (ORR) was defined as the proportion of patients who achieved complete or partial response, and the disease control rate (DCR) was defined as the proportion of patients who achieved complete, partial, or stable disease. Acute toxicities, including mucositis, dermatitis, dysphagia, xerostomia, and hematologic parameters, were graded weekly during treatment and up to 90 days after completion using the Common Terminology Criteria for Adverse Events (CTCAE) version 5.0 [26]. Late toxicities were assessed >90 days after treatment completion.

Overall survival (OS) was defined as the time from the initiation of radiotherapy to death from any cause. Progression-free survival (PFS) was defined as the time from the initiation of radiotherapy to the first documented local, regional, or distant recurrence or death from any cause. Patients who were alive without progression at the time of the last follow-up were censored. Survival curves were estimated using the Kaplan-Meier method.

Sample size

The sample size was calculated for the primary endpoint of tumor response using a one-sample proportion test [27]. Based on published studies reporting response rates of approximately 40-55% in patients with locally advanced oral cavity cancers treated with definitive radiotherapy or chemoradiotherapy [18], a historical response rate of 50% was assumed. The sample size estimation was performed with a two-sided alpha (α) of 0.05 and a power of 80% (β = 0.20). Considering an anticipated 10% non-evaluable rate, the planned recruitment target was 52 patients, and, ultimately, 55 patients were enrolled.

Statistical analysis

All data were entered into prospectively maintained databases. Descriptive statistics were used to summarize the baseline characteristics, treatment details, and toxicity profiles. Continuous variables are reported as medians with ranges, and categorical variables as frequencies and percentages. Tumor responses were summarized using proportions. Survival outcomes (OS and PFS) were calculated from the date of radiotherapy initiation to the event of interest and analyzed using the Kaplan-Meier method. The median survival times with 95% confidence intervals (CIs) were estimated. Patients without events at the last follow-up were censored. As this was a prospective study, missing data were minimal and were handled using complete case analysis. Statistical analyses were performed using the SPSS Statistics software version 25.0 (IBM Corp., Armonk, NY, USA).

## Results

Patient and tumor characteristics

A total of 55 patients with locally advanced OSSCC were enrolled in this prospective study. The median age was 53 years (range = 29-73 years). The gender distribution was nearly equal, with 28 (50.9%) males and 27 (49.1%) females. Most patients (85.5%) had an ECOG performance status of 1. The most common risk factors were betel nut chewing (60%), smoking (21.8%), and alcohol consumption (18.2%). In total, 14 (25.5%) patients had comorbidities, most commonly hypertension (9.1%) and diabetes mellitus (7.3%).

The tongue was the most frequent primary site (40%), followed by the buccal mucosa (21.8%), alveolus (14.5%), gingivobuccal sulcus (16.4%), and hard palate (7.3%). Most tumors were well-differentiated SCCs (56.4%), with 43.6% being moderately differentiated SCCs. At presentation, 41.8% had T4a disease and 21.8% had T4b disease. Nodal status was N2 in 40% and N3 in 21.8% of patients, with nearly 90% presenting with stage IV disease (IVA/IVB). The most frequent reasons for inoperability were fixed nodal disease (21.8%), high masticator space or base of skull involvement (21.8%), and involvement of the oropharynx or floor of mouth (18.2%); 16.4% declined surgery.

Treatment characteristics

Overall, 15 (27.3%) patients received radiotherapy alone, whereas 40 (72.7%) received concurrent cisplatin-based chemotherapy. Most patients completed one to two cycles of cisplatin according to the protocol. The median overall treatment time (OTT) was 43 days (range = 40-55 days). All VMAT plans achieved ≥95% coverage of the PTV by the prescribed dose with acceptable conformity and homogeneity, a maximum dose ≤107%, and adherence to QUANTEC organ-at-risk dose constraints, confirming consistent plan quality across the cohort. The baseline patient, tumor, and treatment characteristics are summarized in Table 1.

**Table 1:** Baseline patient and tumor treatment characteristics (N = 55). ECOG = Eastern Cooperative Oncology Group; SCC = squamous cell carcinoma; OTT = overall treatment time

Characteristic	n (%) or median (range)
Age (years); Median (range)	53 (29–73)
Gender; n (%)	
Male	28 (50.9%)
Female	27 (49.1%)
ECOG performance status; n (%)	
0	8 (14.5%)
1	47 (85.5%)
Risk factors/habits (multiple responses possible); n (%)	
Betel nut chewing	33 (60.0%)
Smoking	12 (21.8%)
Alcohol use	10 (18.2%)
Comorbidities (overall); n (%)	
No	41 (74.5%)
Yes	14 (25.5%)
Comorbidity details (multiple responses possible); n (%)	
Hypertension	5 (9.1%)
Diabetes mellitus	4 (7.3%)
Ischemic heart disease	2 (3.6%)
Post history of tuberculosis	2 (3.6%)
Chronic kidney disease	1 (1.8%)
Primary tumor site; n (%)	
Tongue	22 (40.0%)
Buccal mucosa	12 (21.8%)
Alveolus	8 (14.5%)
Gingivobuccal sulcus	9 (16.4%)
Hard palate	4 (7.3%)
SCC; n (%)	
Well-differentiated SCC	31 (56.4%)
Moderately differentiated SCC	24 (43.6%)
Tumor (T) stage; n (%)	
T2–T3	20 (36.4%)
T4a	23 (41.8%)
T4b	12 (21.8%)
Nodal (N) stage; n (%)	
N0–N1	21 (38.2%)
N2	22 (40.0%)
N3	12 (21.8%)
Reasons for inoperability (multiple responses possible); n (%)	
Fixed node	12 (21.8%)
High masticator space/base of skull involvement	12 (21.8%)
Extensive disease – difficult reconstruction	9 (16.4%)
Involving the oropharynx/floor of the mouth	10 (18.2%)
Patient unwillingness	9 (16.4%)
Chemotherapy received; n (%)	
No	15 (27.3%)
Yes (cisplatin-based)	40 (72.7%)
OTT; median days (range)	43 days (40–55)

Tumor response

At three months post-treatment, the CR rate was 40.0% (n = 22), PR was 38.2% (n = 21), SD was 12.7% (n = 7), and PD was 9.1% (n = 5). The ORR (CR + PR) was 78.2% (95% CI = 65.5-87.3; standard error (SE) = 5.7%, and the DCR (CR + PR + SD) was 90.9% (95% CI = 81.8-98.2; SE = 3.9%). The tumor response data are presented in Table 2.

**Table 2:** Tumor response and acute toxicities (including grade distribution).

Parameter	n (%)
Tumor response	
Complete response	22 (40 %)
Partial response	21 (38.2%)
Stable disease	7 (12.7%)
Progressive disease	5 (9.1%)
Acute toxicities	
Mucositis	
Grade 0–2	27 (49.1%)
Grade ≥3	28 (50.9%)
Dermatitis	
Grade 0–2	44 (80.0%)
Grade ≥3	11 (20.0%)
Dysphagia	
Grade 0–2	41 (74.5%)
Grade ≥3	14 (25.5%)
Xerostomia	
Grade 0–2	55 (100%)
Grade ≥3	0
Anemia	
Grade 0–2	49 (89.1%)
Grade ≥3	6 (10.9%)
Leukopenia	
Grade 0–2	46 (83.6%)
Grade ≥3	9 (16.4%)
Neutropenia	
Grade 0–2	51 (92.7%)
Grade ≥3	4 (7.3%)

Acute toxicities

All patients experienced some degree of oral mucositis, with 28 (50.9%) developing grade ≥3 severity. Dermatitis was nearly universal, but severe cases were less common, with 11 (20.0%) patients experiencing grade ≥3 dermatitis. Dysphagia occurred in most patients, with 14 (25.5%) experiencing grade ≥3 severity, whereas xerostomia was universal but limited to grade ≤2.

Among the hematologic toxicities, grade ≥3 anemia occurred in six (10.9%) patients, grade ≥3 leukopenia in nine (16.4%), and grade ≥3 neutropenia in four (7.3%). These events were predominantly observed in patients who received concurrent cisplatin-based chemotherapy. The acute toxicity profiles are presented in Table 2.

Survival outcomes

**Figure 1:**
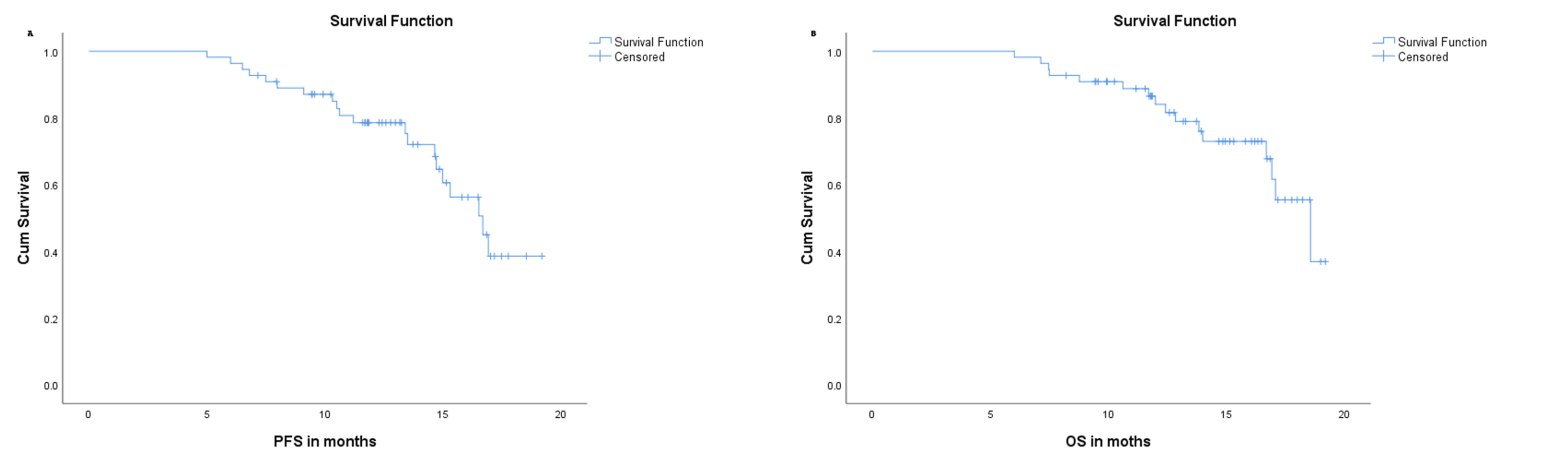
Kaplan-Meier survival curves of patients with locally advanced oral cavity squamous cell carcinoma treated with definitive volumetric modulated arc therapy. (A) Progression-free survival (PFS). (B) Overall survival (OS).

At a median follow-up of 13.4 months (range = 11.5-16.7 months), 20 progression events and 16 deaths occurred among the 55 patients. The median PFS was 16.7 months (95% CI = 14.9-18.5), and the median OS was 18.6 months (95% CI = 16.0-21.2). The estimated one-year PFS and OS rates were approximately 78% (95% CI = 67-89; SE = 5.6%) and 80% (95% CI = 69-91; SE = 5.2%), respectively. The Kaplan-Meier curves for PFS and OS are presented in Figure 1.

## Discussion

This prospective, single-center study evaluated the early clinical outcomes of 55 patients with locally advanced, inoperable OCSCC treated with definitive VMAT, with or without concurrent cisplatin-based chemotherapy. To our knowledge, this represents one of the first prospective Indian datasets focusing exclusively on OCSCC treated with VMAT in a definitive setting.

Treatment response

At three months post-treatment, our cohort demonstrated a CR of 40%, PR of 38.2%, and DCR (CR+PR+SD) of 90.9%. The ORR (78.2%) compares favorably with that of Indian retrospective series, including Mishra et al. (CR = 49%, PR = 25%, ORR = 74%) [18] and Padma et al. [15]. These findings indicate that VMAT-based definitive chemoradiation can achieve at least equivalent, if not superior, short-term tumor control compared with older techniques.

Treatment-related toxicity

Despite the improved conformality of VMAT, acute mucosal toxicities remain a challenge. All patients developed some degree of oral mucositis, with 50.9% experiencing grade ≥3 severity. Dermatitis was nearly universal, but severe cases were less frequent (20% grade ≥3). Dysphagia occurred in most patients, with 25.5% experiencing grade ≥3 severity, whereas xerostomia was universal but limited to grade ≤2. Hematologic toxicities were lower than those reported in many Western studies, with grade ≥3 anemia in 10.9%, leukopenia in 16.4%, and neutropenia in 7.3%. These rates are broadly comparable to earlier Indian studies [15] and align with Scher et al. [14] for mucositis and dysphagia, while our hematologic toxicity profile likely reflects the stringent eligibility criteria and proactive supportive care.

Survival outcomes

At a median follow-up of 13.4 months, the median PFS was 16.7 months (95% CI = 14.9-18.5) and the median OS was 18.6 months (95% CI = 16.0-21.2), with one-year PFS of 78% and one-year OS of 80%. These early outcomes compare well with other Indian and international retrospective series, including those by Shenoy et al. [19], Elbers et al. [16], and Patil et al. [17], who have generally reported median OS below 15-16 months or questioned the efficacy of chemoradiation in unresectable oral cavity cancer. Western series such as Scher et al. [14] also describe substantially poorer long-term outcomes (two-year OS of ~30-35%), reflecting differences in case mix, treatment techniques, and follow-up duration. Because our median follow-up was short, these survival estimates should be interpreted as early control rates rather than definitive benchmarks.

Our median OTT of 43 days compares favorably with other Indian series [15,17,19], where OTT frequently exceeds 45-50 days. Maintaining OTT is crucial, as treatment prolongation is associated with reduced locoregional control and survival [7,11]. High treatment compliance in our cohort likely contributed to favorable early outcomes.

Taken together, these findings reinforce that VMAT is feasible and effective for delivering definitive chemoradiation in OCSCC. Compared with two-dimensional/three-dimensional conformal radiotherapy and step-and-shoot IMRT, VMAT allows more conformal coverage, better sparing of normal structures, and faster treatment delivery [5,6]. This degree of precision makes definitive chemoradiation more tolerable in a setting where high-dose treatment would likely have been prohibitively toxic with older techniques. Nevertheless, even with VMAT, severe mucositis and dysphagia remain common because of the unavoidable high doses to the oral mucosa [8,10,11].

From a practical standpoint, the adoption of VMAT in resource-limited centers may be constrained not by the availability of treatment units but by software licensing expenses and the requirement for adequately trained planning staff. Although VMAT planning demands greater computational and human resources than three-dimensional conformal radiotherapy or step-and-shoot IMRT, it operates on the same linear accelerator infrastructure already used for advanced radiotherapy. Once implemented, VMAT enhances overall efficiency through faster treatment delivery, reduced machine time, and fewer interruptions. These advantages make it a pragmatic and sustainable option for high-volume South Asian oncology centers striving to balance precision, efficiency, and affordability within existing healthcare resources.

Strengths and limitations

The key strengths of this study include its prospective design, uniform VMAT delivery, and standardized response and toxicity assessments using RECIST v1.1 and CTCAE v5.0. Limitations include the relatively short median follow-up, which restricts the assessment of late toxicities and long-term survival, and its single-center nature, which may affect generalizability. A small subset of patients declined surgery despite borderline resectability, as predefined in the eligibility criteria, reflecting real-world, patient-centered practice rather than selection bias. Continued follow-up and multi-institutional validation are required to establish evidence-based benchmarks for VMAT in this setting.

## Conclusions

In this prospective, single-center study of patients with unresectable OCSCC who had good performance status, non-surgical treatment with VMAT-based chemoradiotherapy was feasible, yielding effective and acceptable early outcomes. These findings support modern radiotherapy as a viable option when surgery is not possible; however, a longer follow-up is required to assess late toxicities and long-term survival.
